# Self-Healing Hydrogels with Intrinsic Antioxidant and Antibacterial Properties Based on Oxidized Hydroxybutanoyl Glycan and Quaternized Carboxymethyl Chitosan for pH-Responsive Drug Delivery

**DOI:** 10.3390/gels11030169

**Published:** 2025-02-26

**Authors:** Jae-pil Jeong, Kyungho Kim, Eunkyung Oh, Sohyun Park, Seunho Jung

**Affiliations:** 1 Department of Bioscience and Biotechnology, Microbial Carbohydrate Resource Bank (MCRB), Konkuk University, 120 Neungdong-ro, Gwangjin-gu, Seoul 05029, Republic of Korea; jjp0531@naver.com (J.-p.J.); rudgh971225@naver.com (K.K.); eunkyung_5@naver.com (E.O.); so63991@naver.com (S.P.); 2 Department of System Biotechnology, Microbial Carbohydrate Resource Bank (MCRB), Konkuk University, 120 Neungdong-ro, Gwangjin-gu, Seoul 05029, Republic of Korea

**Keywords:** oxidized 3-hydroxybutanoyl glycan (OHbG), quaternized carboxymethyl chitosan(QCMCS), hydrogel, pH-responsive drug delivery, antibacterial activity, antioxidant efficiency

## Abstract

In this study, self-healing hydrogels were created using oxidized hydroxybutanoyl glycan (OHbG) and quaternized carboxymethyl chitosan (QCMCS), displaying antioxidant and antibacterial properties for pH-responsive drug delivery. The structures of the modified polysaccharides were confirmed through ^1^H NMR analysis. Double crosslinking in the hydrogel occurred via imine bonds (between the aldehyde group of OHbG and the amine group of QCMCS) and ionic interactions (between the carboxyl group of OHbG and the quaternized group of QCMCS). The hydrogel exhibited self-healing properties and improved thermal stability with an increase in OHbG concentration. The OHbG/QCMCS hydrogel demonstrated high compressive strength, significant swelling, and large pore size. Drug release profiles varied between pH 2.0 (96.57%) and pH 7.4 (63.22%). Additionally, the hydrogel displayed antioxidant and antibacterial effects without compromising the polysaccharides’ inherent characteristics. No cytotoxicity was observed in any hydrogel samples. These findings indicate that the OHbG/QCMCS hydrogel is a biocompatible and stimuli-responsive drug carrier, with potential for various pharmaceutical, biomedical, and biotechnological applications.

## 1. Introduction

Drug delivery systems are effective in treating various human diseases in the biomedical field, highlighting the importance of developing suitable drug carriers [1,2]. These carriers are available in various forms, including liposomes, nanoparticles, hydrogels, and films [3,4]. Among these systems, hydrogels are defined as 3D network structures formed through non-covalent or covalent crosslinking methods [5,6]. Non-covalent bonding crosslinking methods include ionic bonding, hydrogen bonding, hydrophobic interactions, and ultrasonic sonication, whereas covalent crosslinking involves organic reactions like click chemistry, Michael addition, and Schiff base formation [7]. To enhance the properties and mechanical strength of hydrogel networks, researchers have explored multi-crosslinked hydrogels by combining two or more crosslinking methods [8,9,10]. In the case of multi-crosslinked hydrogels, the self-healing ability has been reported due to various non-covalent and dynamic covalent interactions [11,12]. Non-covalent interactions include electrostatic interactions between positive and negative charges, network formation through metal chelation, and host–guest interactions. Dynamic covalent bonds include Schiff base bonds, disulfide bonds, and borate ester bonds. Among various hydrogel network types, Schiff base-crosslinked hydrogels have been extensively studied for their pH-responsive properties [13,14]. Under acidic conditions, imine bonds dissociate in response to pH changes, enabling the controlled release of specific drugs in a pH-dependent environment change [15]. Additionally, as this reaction is reversible, Schiff base-crosslinked hydrogels have also been reported to exhibit self-healing properties [16,17]. The imine bond of a Schiff base undergoes protonation in a low pH environment, which promotes hydrolysis and leads to the degradation of the crosslinking. However, under neutral and alkaline pH conditions, the imine bond remains stable and can reversibly form.

Hydrogel networks are usually made from natural or synthetic polymers. Naturally derived polymers include polysaccharides, proteins, and lipids, which are often selected as hydrogel backbones because they have lower toxicity, greater biocompatibility, and faster biodegradation rates than synthetic polymers [18,19]. Polysaccharides are particularly recognized as ideal candidates for fabricating hydrogels due to their diverse functionalities, molecular weights, and excellent biocompatibility. Furthermore, polysaccharides possess anionic, neutral, and cationic properties because of their functional groups, making them advantageous for forming hydrogel networks through electrostatic interactions [20,21]. Polysaccharides can be obtained from animals, plants, fungi, and microorganisms. Recently, microbial polysaccharides have received attention for their high yield, versatile sources, ease of isolation, and tunable chemical composition and structure, making them ideal for pharmaceuticals, medical devices, and cosmetics [22]. Hydrogels typically possess a porous structure resulting from crosslinking, which allows them to effectively deliver drugs within this network. In this case, to maximize drug stability and delivery effectiveness, the inherent properties of the hydrogel can play a vital role in its application as a candidate for drug delivery systems, such as its antioxidant, antibacterial, anti-inflammatory functionalities [23,24].

Rhizobia, Gram-negative soil bacteria, interact with legume hosts. Under nitrogen-limiting conditions, they induce nodule formation on plant roots, where they invade and colonize, converting dinitrogen to ammonia for plant use. Exopolysaccharides released from rhizobia are crucial for establishing effective symbiotic nodulation [25,26]. In addition to this function, rhizobial exopolysaccharides have been reported to display biological effects, including antioxidant and anticancer activities, as well as high thermal stability [27,28]. Hydroxybutanoyl glycan (HbG) is a rhizobial anionic exopolysaccharide composed of five D-glucose units, two D-glucuronic acids, and one D-galactose. These sugar units are connected by α-1,4, β-1,3, β-1,4, and β-1,6 glycosidic linkages. Previous studies have demonstrated that HbG exhibits antioxidant properties due to its uronic acid content [29]. Recently, studies have been reported on polysaccharide-based dialdehyde derivatives synthesized through periodate oxidation [19,20]. These derivatives successfully formed hydrogels via Schiff base interactions with amine-containing polymers [30,31].

Chitosan (CS) is a linear polysaccharide composed of randomly distributed β-(1→4)-linked D-glucosamine and N-acetyl-D-glucosamine units. CS has gained attention due to its abundance and biological effects, such as antibacterial activity, biocompatibility, and biodegradability [32,33]. However, CS is soluble only under acidic conditions, and its strong intra- and inter-molecular hydrogen bonding results in low mechanical properties [34]. To overcome these limitations, chemical modifications of CS have been extensively studied to increase water solubility under neutral conditions and enhance mechanical strength [35]. Carboxymethyl chitosan (CMCS), the most widely used CS derivative, is synthesized by reacting the primary alcohol group of CS with monochloroacetic acid. CMCS has demonstrated antioxidant and anticancer effects, in addition to the inherent antibacterial properties of CS, making it widely used in cosmetic and biomedical fields [36]. Quaternized chitosan (QCS) is another derivative, where a quaternary ammonium group is introduced into the amine group of CS. QCS is highly soluble under both acidic and basic conditions and exhibits potent antioxidant and antibacterial activities [37,38]. Recent studies have explored the synergistic effects of carboxymethylation and quaternization, resulting in quaternized carboxymethyl chitosan (QCMCS), which exhibits enhanced solubility, biocompatibility, and bacteriostatic properties [39,40].

In this study, we fabricated hydrogels composed of oxidized hydroxybutanoyl glycan (OHbG) and QCMCS. We hypothesized that (1) the prepared hydrogels would possess strong antioxidant and antibacterial activities due to the carboxyl groups in OHbG, which contain glucuronic acid and pyruvate, and the cationic functional groups (amine and quaternized) in QCMCS; (2) the hydrogels would exhibit self-healing properties due to the Schiff base and ionic interactions within the network, as well as improved thermal stability; and (3) the swelling rate and degradation would vary with pH, resulting from the Schiff base and ionic interactions. To test this hypothesis, we conducted NMR and FTIR analyses to confirm the structure of the modified polysaccharides. In addition, we performed thermal and rheological analyses to verify their enhanced mechanical strength and thermal stability. Also, 5-Fluorouracil (5-FU) was selected as a model drug for investigating the drug release. 5-FU is a hydrophilic anticancer drug primarily used in the treatment of gastrointestinal cancers and breast cancer, and it is one of the most widely used antimetabolite drugs in first-line therapy [41,42]. Drug release studies using 5-fluorouracil under different pH conditions were conducted to assess the pH-responsive behavior of the hydrogels. Finally, their antioxidant and antibacterial properties were evaluated to determine whether the inherent functionality of each polysaccharide was preserved.

## 2. Results and Discussion

### 2.1. Nuclear Magnetic Resonance Spectroscopy (NMR) Analysis

**Figure 1:**
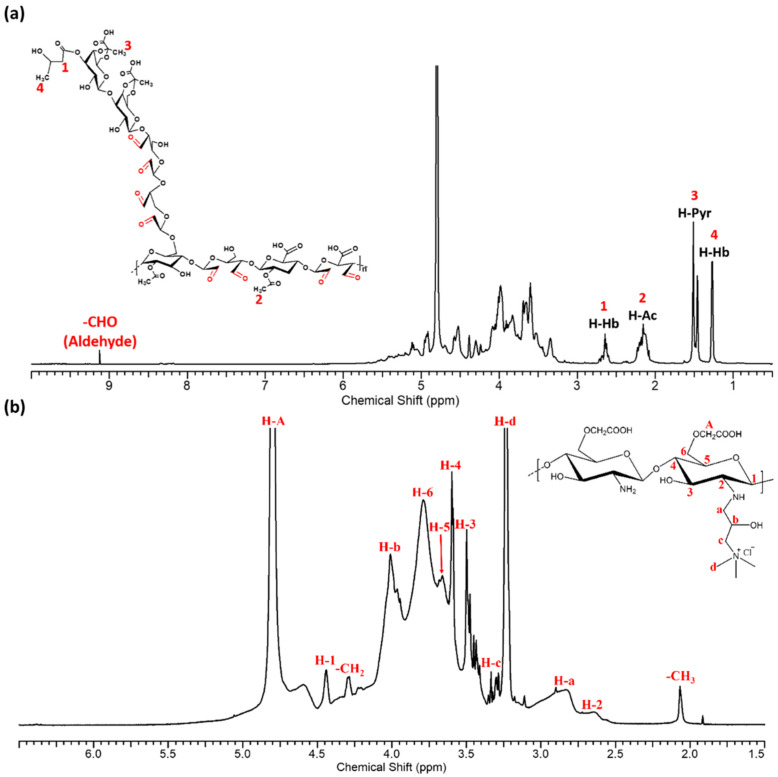
^1^H NMR spectrum of (**a**) oxidized 3-hydroxylbutanoyl glycan (OHbG) and (**b**) quaternized carboxymethyl chitosan (QCMCS).

OHbG was obtained from HbG via the NaIO_4_-mediated oxidation of the vicinal glycol groups in the polysaccharide. HbG contained one 3-hydroxybutanoyl group, two acetyl groups, and one pyruvyl group in its octasaccharide repeating unit. In the ^1^H NMR spectrum of HbG, the peaks corresponding to each functional group were at 2.61 ppm (1, H-Hb), 2.16 ppm (2, H-Ac), 1.48 ppm (3, H-Pyr), and 1.28 ppm (4, H-Hb) [43,44]. Following the oxidation process, the primary difference observed in OHbG was the introduction of an aldehyde group, which was evidenced by the appearance of a characteristic peak at 9.12 ppm. The degree of oxidation was calculated to be 91.45 ± 0.19% (Figure 1a).

The ^1^H NMR spectrum of QCMCS showed that the quaternary ammonium group was grafted successfully onto carboxymethyl chitosan through the previous method. The peaks at 4.44 ppm (H-1), 2.65 ppm (H-2), 3.49 ppm (H-3), 3.59 ppm (H-4), 3.68 ppm (H-5), and 3.79 ppm (H-6) correspond to the backbone structure of QCMCS. The protons of the methyl group in the quaternary ammonium group appeared at 3.24 ppm (H-d), while additional peaks at 2.88 ppm (H-a), 4.01 ppm (H-b), and 3.34 ppm (H-c) further confirmed the grafting of the quaternary ammonium groups onto CMCS. A peak at 4.79 ppm was attributed to the protons in the carboxymethyl groups [45,46] (Figure 1b).

### 2.2. FTIR Analysis

**Figure 2:**
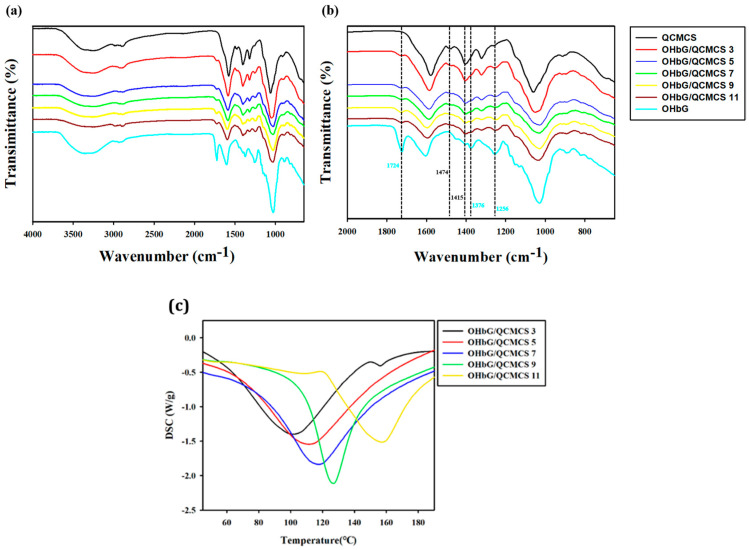
(**a**) FTIR spectra from 4000 to 650 cm^−1^ of OHbG/QCMCS hydrogels. (**b**) FTIR spectra from 2000 to 650 cm^−1^ of OHbG/QCMCS hydrogels. (**c**) DSC curves of OHbG/QCMCS hydrogels.

The FTIR spectra of the OHbG, QCMCS, and OHbG/QCMCS hydrogels are presented in Figure 2a,b. As previously reported, the characteristic peaks of OHbG were observed at 1724 cm^−1^, 1376 cm^−1^, and 1256 cm^−1^, corresponding to the C=O stretching of the carbonyl ester group from the acetyl group, the symmetric stretching vibration of the carboxylate (COO^−^) group from acid residues, and the C–O stretching of the ester linkage in the 3-hydroxybutanoyl group, respectively [29]. For QCMCS, a distinct absorption band at 1474 cm^−1^ was assigned to the C–H bending vibrations, confirming the introduction of the quaternary ammonium group in chitosan [47,48]. The characteristic peaks of both OHbG and QCMCS were modulated based on their concentration in the hydrogels. As the proportion of OHbG increased, the intensity of the OHbG peaks became stronger, while the QCMCS peaks diminished in intensity. Additionally, slight shifts in the peaks of amine group (-NH_2_, 1415–1400 cm^−1^) were observed, likely due to interactions between OHbG and QCMCS within the hydrogel matrix [49]. Before crosslinking, QCMCS exhibited a distinct amine group peak at 1415 cm^−1^ with strong intensity. However, as the proportion of OHbG increased in the hydrogel, the intensity of the amine group peak gradually decreased.

### 2.3. Differential Scanning Calorimetry (DSC)

The thermal stability of the OHbG/QCMCS hydrogel network was evaluated through DSC analysis. The results (Figure 2c) showed endothermic peaks at 102.15 °C, 111.89 °C, 117.81 °C, 126.55 °C, and 157.59 °C for OHbG/QCMCS 3, 5, 7, 9, and 11. The DSC curves showed that an increase in OHbG concentration raised the endothermic peak temperature from 102.15 °C to 157.59 °C. These enhanced thermal stabilities were attributed to the increase in molecular interactions, such as hydrogen bonding, imine bonding, and van der Waals forces, due to higher OHbG concentration. These findings align with previous studies indicating that OHbG enhanced hydrogel thermal stability [50,51].

### 2.4. Rheological Analysis

Rheological measurements were performed to assess the physical stiffness of hydrogel. This method is commonly used to characterize hydrogels’ mechanical strength and viscoelastic behavior [52,53,54]. Rheological measurements of hydrogels were performed to analyze the mechanical properties of the OHbG/QCMCS hydrogels depending on the concentration of OHbG. An angular frequency sweep test measured the physical stiffness, showing the highest storage modulus (G’) and loss modulus (G”) values in OHbG/QCMCS 11. This indicates that the results increased with higher OHbG ratios. The G’ values were 1011 Pa, 1402 Pa, 3404 Pa, 3715 Pa, and 5584 Pa for OHbG/QCMCS 3, 5, 7, 9, and 11. An increase in OHbG concentration led to the greater polymer contribution to the hydrogel structure, enhancing physical stiffness.

**Figure 3:**
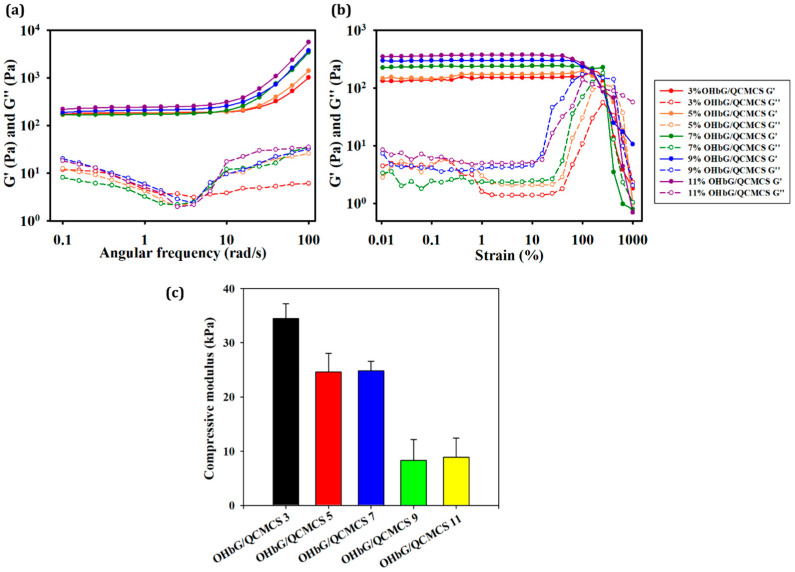
(**a**) Angular frequency sweep test (0.1 rad/s to 100 rad/s) of OHbG/QCMCS hydrogels at a constant strain (1%). (**b**) Oscillation strain amplitude sweep test (from 0.1% to 1000%) of OHbG/QCMCS hydrogels at a constant angular frequency (1 Hz). (**c**) Compressive stress–strain test of OHbG/QCMCS hydrogels.

Amplitude strain sweep tests assessed the flexibility of each OHbG/QCMCS hydrogel. Figure 3b shows the crossover points of G′ and G′′ for the hydrogels at 415%, 316%, 260%, 180%, and 163% for OHbG/QCMCS 3, 5, 7, 9, and 11, respectively. The decreasing crossover point values at higher OHbG concentrations indicated that an increase in OHbG enhanced the interaction points within the hydrogel network, reducing flexibility following the OHbG ratio.

### 2.5. Compressive Test

Compressive tests were conducted to evaluate and compare the mechanical strength of each OHbG/QCMCS hydrogel. Figure 3c shows the variation in compressive modulus with OHbG concentration. The measured compressive modulus values were 34.49 kPa, 24.6 kPa, 24.83 kPa, 8.33 kPa, and 8.91 kPa for OHbG/QCMCS 3, 5, 7, 9, and 11 (*n* = 4, *p* < 0.001). These results indicate that OHbG/QCMCS 11 was the most rigid, while OHbG/QCMCS 3 was the most flexible, with OHbG acting as a crosslinker and enhancing bonding within the network. From the rheological measurements, it was observed that an increase in OHbG content led to enhanced solid-like elasticity, while simultaneously resulting in a more rigid and less flexible hydrogel structure [55]. Rigid hydrogels offer a promising solution for addressing stiffness mismatches between hydrogels and human tissues, especially in multi-material 3D printing [56]. In contrast, flexible hydrogels offer a soft and adaptive layer, making them beneficial for wound healing applications that require versatility and gentleness [57,58]. Therefore, the tunable mechanical properties of OHbG/QCMCS hydrogels make them suitable for various applications.

### 2.6. Self-Healing Ability of OHbG/QCMCS Hydrogels

**Figure 4:**
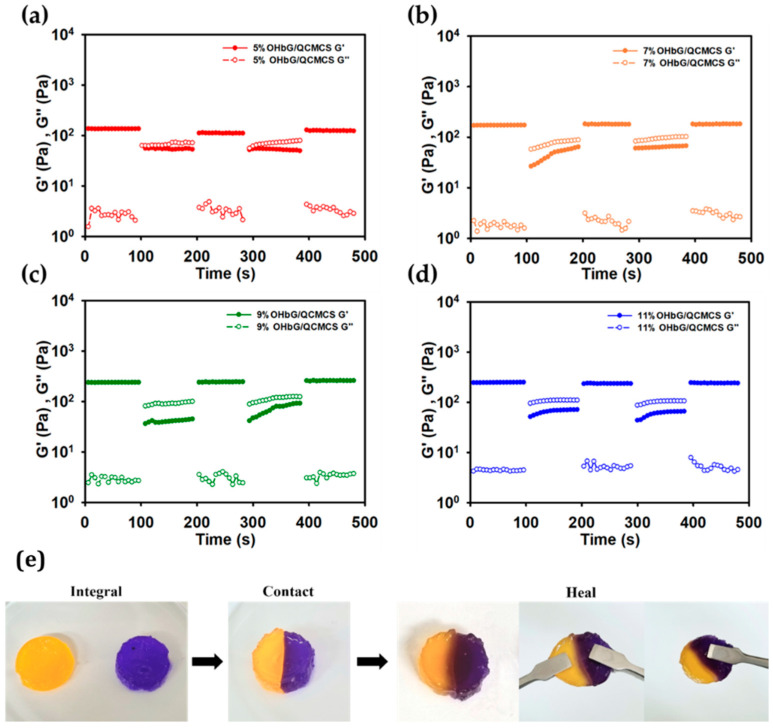
Self-healing ability via the step strain measurements of OHbG/QCMCS hydrogels. (**a**) OHbG/QCMCS 5, (**b**) OHbG/QCMCS 7, (**c**) OHbG/QCMCS 9, and (**d**) OHbG/QCMCS 11. (**e**) Photo of self-healed OHbG/QCMCS hydrogel through the integration of two cleaved hydrogels with different colors.

Cyclic continuous step strain tests were conducted to assess the self-healing properties of OHbG/QCMCS hydrogels (Figure 4a–d). The hydrogels experienced 500% strain under a frequency of 1.0 Hz. After being subjected to 500% strain for 100 s, the strain was reduced to 0.5% for an additional 100 s. Figure 4a–d shows that during the application of 500% strain, the G′ and G′′ values reversed, indicating a gel-to-sol transition. However, upon reducing strain to 0.5%, G′ recovered, showcasing the hydrogel’s ability to regain its original strength. This rapid recovery was attributed to the dynamic reorganization of the hydrogel network from Schiff bases [59]. The recovery efficiencies were 82.07%, 93.38%, 94.28%, and 96.04% for OHbG/QCMCS 5, 7, 9, and 11, with OHbG/QCMCS 11 showing the highest recovery efficiency due to the increased number of interaction points in the hydrogel network. Figure 4e further demonstrates the self-healing properties of the OHbG/QCMCS hydrogel by showcasing the successful integration of two distinctly colored hydrogels.

### 2.7. Swelling and Degradation Profiles of OHbG/QCMCS Hydrogels

**Figure 5:**
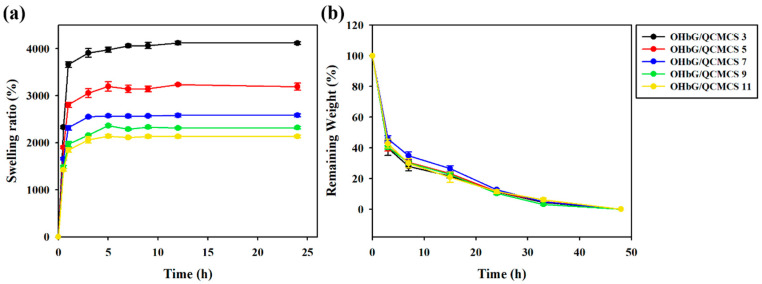
(**a**) Swelling ratio of OHbG/QCMCS hydrogels at physiological conditions (pH 7.4) and (**b**) the percentage of the remaining weight of OHbG/QCMCS hydrogels at acidic conditions (pH 2.0).

The swelling capacity of the OHbG/QCMCS hydrogels was assessed in PBS buffer (pH 7.4) at 37 °C. Figure 5a shows that all hydrogels displayed rapid swelling, reaching equilibrium within 5 h. At equilibrium, the swelling ratio decreased with higher OHbG concentrations, which corresponds to the increased crosslinking within the hydrogels [60,61]. As the OHbG concentration increased, crosslinking between QCMCS and OHbG also increased, leading to a denser network and reduced swelling capacity. The degradation properties of the OHbG/QCMCS hydrogels were assessed in PBS buffer (pH 2.0) at 37 °C. The initial weights of the hydrogel samples are denoted in Table S1.

Degradation was monitored after the hydrogels reached their maximum swollen state, as further swelling was not possible under these conditions [62]. Figure 5b shows that all hydrogel samples were completely degraded after 48 h. Although Schiff base interactions are stable at physiological pH (7.4), hydrolysis occurred under acidic conditions (pH 2.0), explaining the degradation of the OHbG/QCMCS hydrogels in such environments [63,64]. These results indicated that OHbG/QCMCS hydrogels could be customized for various applications, with the properties being adjustable by altering conditions such as crosslinker concentration and pH.

### 2.8. Morphology Analysis of OHbG/QCMCS Hydrogels

**Figure 6:**
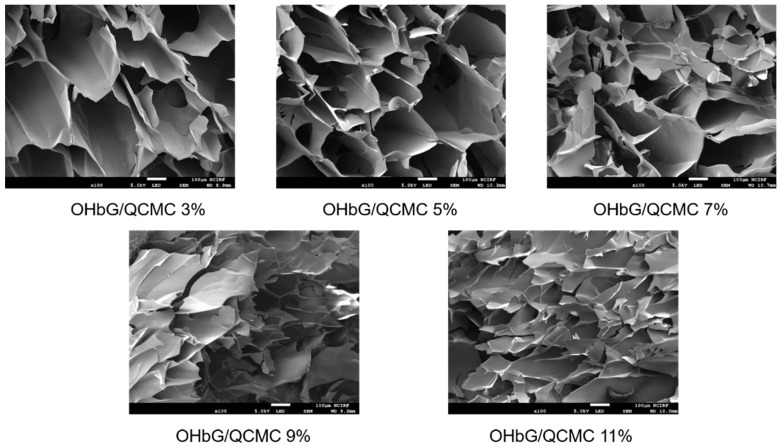
SEM image of OHbG/QCMCS hydrogels. The white scale bar means 100 μm.

To investigate the hydrogel network’s morphology, SEM analysis was conducted on each sample. Figure 6 shows that the pores of all hydrogels are clearly visible. As the OHbG ratio increased, the pore size decreased, indicating a more compact and dense network of OHbG and QCMCS. This finding aligned with the swelling ratio data, as a tighter hydrogel network limited the space for water molecules to penetrate. Hydrogels with larger pores displayed higher swelling rates due to the quicker diffusion of water molecules into the hydrogel [65,66]. The compressive test further supported this finding by demonstrating that as the OHbG content increased, the free space within the OHbG/QCMCS hydrogel decreased. Consequently, the hydrogel became more rigid and less flexible [67].

### 2.9. Drug Release Pattern of OHbG/QCMCS Hydrogels

**Figure 7:**
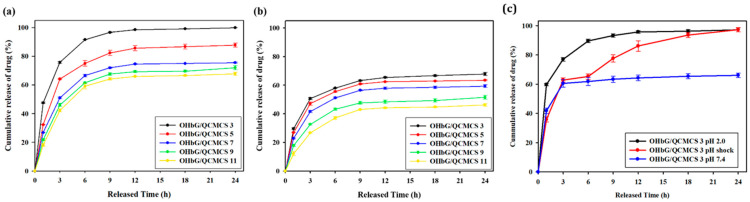
pH-dependent drug release of OHbG/QCMCS hydrogels: cumulative drug release of 5-FU under (**a**) pH 2.0 at 37 °C, (**b**) pH 7.4 at 37 °C, (**c**) pH shock from 7.4 to 2.0. In the pH shock test, the pH was adjusted to 2.0 at 9 hours after the release began at an initial pH of 7.4.

The in vitro drug release profiles of OHbG/QCMCS hydrogels were studied using 5-fluorouracil (5-FU). Prior to the release tests, a standard curve for 5-FU was generated by measuring its absorbance at 266 nm. Each hydrogel was loaded with 1 mg of 5-FU for the experiment. Figure 7a,b presents the cumulative, pH-dependent drug release patterns in PBS buffer at pH 7.4 and 2.0, showing their significant differences under varying pH conditions. At pH 2.0 (Figure 7a), the releasing amounts of the drug were 96.57%, 82.30%, 71.91%, 67.55%, and 64.16% for OHbG/QCMCS 3,5,7,9, and11. In contrast, at pH 7.4 (Figure 7b), the releasing amounts of the drug were 63.22%, 60.86%, 56.43%, 47.66%, and 42.99% for OHbG/QCMCS 3,5,7,9, and 11. Under pH shock conditions, OHbG/QCMCS 3 was selected for further testing due to its highest release pattern (Figure 7c). The drug was released at pH 7.4 for 6 h, then shifted to pH 2.0, resulting in a rapid increase in drug release under acidic conditions compared to pH 7.4. Within 24 h, the amount released under pH shock matched that seen at pH 2.0. This is because the network structure of the OHbG/QCMCS hydrogel is influenced by acidic pH conditions. The Schiff base bonds dissociate under acidic conditions, and the ionic interactions between carboxyl (-COOH) and amine (-NH₂) groups weaken as the pH decreases [68,69,70]. As a result, the gel network rapidly degrades, accelerating drug release. These patterns indicated that OHbG/QCMCS hydrogels could effectively serve as drug carriers in gastric and intestinal systems due to their pH-responsive drug release behavior [71]. The images of hydrogels after their release are showed in Figure S1.

### 2.10. Antioxidant Activity of OHbG/QCMCS Hydrogels

**Figure 8:**
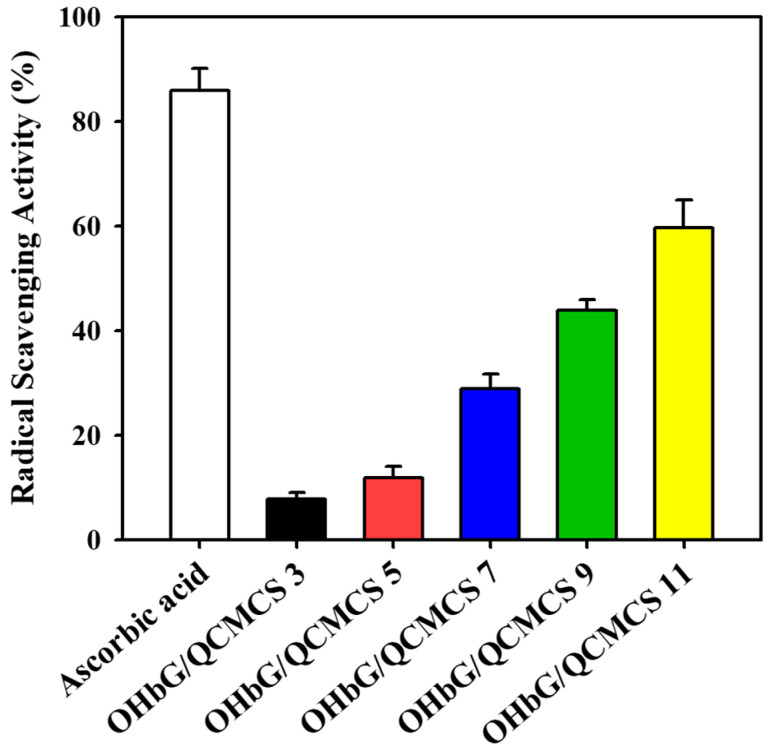
DPPH-scavenging activity of OHbG/QCMCS hydrogels.

The antioxidant capacity of hydrogels is crucial for eliminating reactive oxygen species such as peroxide, superoxide, hydroxyl radicals, and singlet oxygen [72]. Their radical scavenging activity was evaluated using the DPPH radical scavenging method, employing a stable synthetic free radical [73]. Figure 8 illustrates that the antioxidant activity of OHbG/QCMCS hydrogels increased with OHbG concentration. The DPPH radical scavenging activities were 7.67%, 11.78%, 28.79%, 43.82%, and 59.68% for OHbG/QCMCS 3,5,7,9,11. The OHbG/QCMCS samples from 3% to 11% showed antioxidant activities of 7.67 × 10^−4^ µmol/mg, 1.78 × 10^−3^ µmol/mg, 2.88 × 10^−3^ µmol/mg, 4.38 × 10^−3^ µmol/mg, and 5.97 × 10^−3^ µmol/mg, quantitatively. This indicated that OHbG significantly boosts the hydrogels’ antioxidant activity, likely due to the glucuronic acid in the polysaccharide structure. Previous studies have found that the higher uronic acid content in polysaccharides is associated with greater antioxidant activity [74].

### 2.11. Antibacterial Activity of OHbG/QCMCS Hydrogels

**Figure 9:**
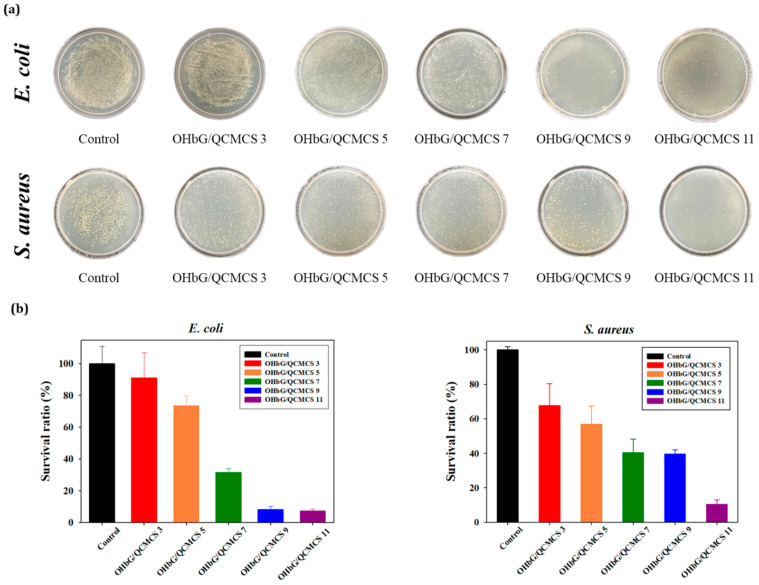
(**a**) Photographs of the antibacterial effect of OHbG/QCMCS hydrogels against *E. coli* and *S. aureus.* (**b**) Survival ratio of OHbG/QCMCS hydrogels against *E. coli* and *S. aureus* (n = 3).

To investigate the antibacterial effects of the hydrogel, *E. coli* and *S. aureus* were selected as representatives of Gram-negative and Gram-positive bacteria. Figure 9 showed that the OHbG/QCMCS hydrogel exhibited antibacterial effects, which increased with higher OHbG contents. The hydrogel with 11% OHbG demonstrated the strongest antibacterial effect, with a 92.7% inhibition against *E. coli* and 89.7% against *S. aureus*. This effect was attributed to the aldehyde groups in the hydrogel matrix. The hydrogel composition was varied by adjusting the OHbG content while keeping the QCMCS amount constant. With QCMCS fixed at 3%, the composition allowed for an increase in unreacted aldehyde groups as the OHbG proportion rose from 3% to 11%. Aldehyde groups are known to be highly reactive, potentially causing bacterial death by damaging cell walls and membranes, interacting with surface amines to disrupt bacterial enzyme systems, or inhibiting substrate contact with enzymes [75,76,77]. Furthermore, the antibacterial effect of the OHbG/QCMCS hydrogel was stronger against *E. coli* than *S. aureus*, attributed to the differences in cell membrane composition between the two bacteria [78].

### 2.12. In Vitro Cytotoxicity Test of OHbG/QCMCS Hydrogels

**Figure 10:**
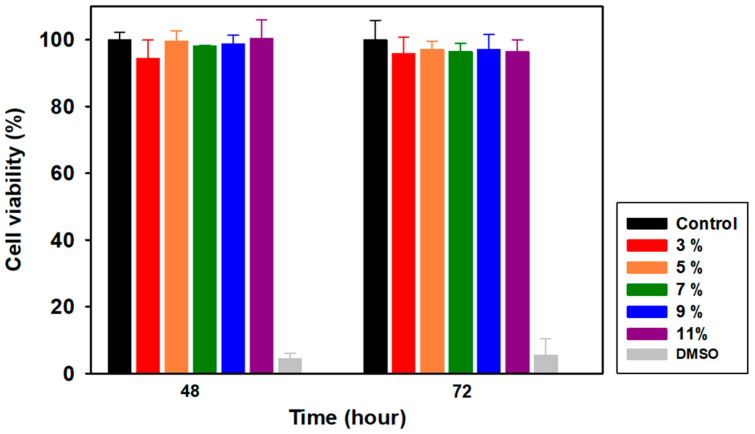
Cytotoxicity test of OHbG/QCMCS hydrogels against HEK-293 cell.

To assess the biocompatibility of the hydrogels, cytotoxicity was evaluated using the MTT assay. The viability rate of HEK 293 cells cultured with OHbG/QCMCS hydrogels was compared to control groups treated with DMEM and DMSO as the negative and positive controls (Figure 10). The results showed that the viability of HEK 293 cells incubated with the hydrogels did not significantly differ from the negative control. The cell viabilities were measured at 94.25%, 99.46%, 97.99%, 98.59%, and 100.26% for 3, 5, 7, 9, and 11% OHbG concentrations after 48 h, and 95.75%, 96.91%, 96.32%, 96.99%, and 96.35% after 72 h, indicating that the OHbG/QCMCS hydrogels exhibited no cytotoxicity.

## 3. Conclusions

The OHbG/QCMCS hydrogels were successfully synthesized through imine bonding and ionic interactions. ^1^H NMR and FTIR analyses confirmed the modification of OHbG and QCMCS structures. The hydrogels demonstrated self-healing properties supported by rheological measurements. Thermal stability increased with added OHbG, reaching a maximum of 157.59 °C. OHbG/QCMCS 3 exhibited the highest elasticity, based on their swelling ratio, compressive strength (34.49 kPa), rheological value (415%), and SEM results. Drug release from OHbG/QCMCS 3 was the highest under both acidic (96.57%) and physiological (63.22%) conditions. The hydrogels maintained biological effects, including antioxidant activity (7.67–59.68%) and antibacterial properties (92.7% against *E. coli* and 89.7% against *S. aureus*). No cytotoxicity was detected, confirming their biocompatibility and safety for medical applications.

Overall, this hydrogel system, with a self-healing capability, pH responsiveness, and antioxidant and antibacterial properties, has significant potential for biomedical applications, particularly in drug delivery and wound healing. Its ability to modulate rapidly responsive drug release based on pH makes it suitable for targeted therapies like gastrointestinal drug delivery. Additionally, its biocompatibility and mechanical strength suggest its potential for applications in tissue engineering and regenerative medicine.

## 4. Materials and Methods

### 4.1. Materials

Glycidyltrimethylammonium chloride (GTMC, technical grade ≥90%), sodium periodate (NaIO_4_, 98.0%), potassium iodide (KI, 99.0%) ascorbic acid (99.7%), 2,2-diphenyl-1-picrylhydrazyl (DPPH, 98%), 5-fluorouracil (99.0%), and LB broth were supplied from Sigma-Aldrich (Steinheim, Germany). All other chemicals were of analytical grade and were used as received, without any additional purification.

### 4.2. Preparation of 3-Hydroxylbutanoyl Glycan (HbG)

*Rhizobium leguminosarum bv.viciae* VF39 was fermented in glutamate-mannitol-salts (GMS) medium at 28 °C for 5 days. The production medium consists of D-mannitol (10 g/L), glutamic acid (1.0 g/L), K_2_HPO_4_ (1.0 g/L), MgSO_4_∙7H_2_O (0.2 g/L), CaCl_2_∙2H_2_O (0.04 g/L), and trace elements, with the pH adjusted to 7.0. Cultured cells were removed by centrifugation with 8000× *g* at 4 °C for 15 min. The supernatant was collected, and three volumes of ethanol were added for precipitation. The precipitate underwent dialysis by a membrane tube (MWCO 12–14 kDa) against distilled water (DW) for 3 days. Washed and purified HbG was lyophilized for further study.

### 4.3. Preparation of Oxidized 3-Hydroxylbutanoyl Glycan (OHbG)

OHbG was prepared via NaIO_4_-mediated oxidation, which was applied to various polysaccharides [50,79]. First of all, 10 g of HbG was suspended in 900 mL of DW at 60 °C. Next, 7.2 g of NaIO_4_ was dissolved in 100 mL of DW. Afterwards, the HbG solution was mixed with the NaIO_4_ solution and stirred at 25 °C for 5 h. The reaction was quenched by adding 2 mL of ethylene glycol to the solution. The precipitate was dialyzed against DW using membrane tubes (MWCO 12–14 kDa) for 3 days. After the dialysis, purified OHbG was lyophilized and kept in a desiccator for later use. The oxidation degree was measured via the iodine titration method [80]. Briefly, 1 mL of the reaction solution was sampled before quenching and used for titration. A total of 0.4 mL of 20% KI solution was added to the reaction mixture in a beaker. Then, 10% starch solution was used as an indicator, and titration was performed using sodium thiosulfate.

### 4.4. Preparation of Quaternized Carboxymethyl Chitosan (QCMCS)

QCMCS was synthesized following a previously reported method [81]. Firstly, 2.18 g of CMCS was dissolved in 100 mL of DW. Next, 0.45 g of GTMC was added and the mixture was reacted at 55 °C for 24 h. The resulting QCMCS was then dialyzed in a membrane tube (MWCO: 12–14 kDa), with DW for 3 days. After the dialysis, the purified QCMCS was lyophilized for further study. The degree of modification was calculated with the integration of NMR data [49]. This was calculated using the following equation.

$$Degree\;of\;Modification\left(QCMCS\right)=\frac{{(I}_{d}/9)\times Degree\;of\;deacetylation}{I_{CH_{3}\;of\;acetyl}/3}\times 100\%$$

$I_{d}$ is the peak of the methyl group at the quaternized ammonium group in 3.2 ppm. $I_{CH_{3}\;of\;acetyl}$ is the peak of the methyl group at the acetyl group in 2.1 ppm. The degree of modification was 51%.

### 4.5. Preparation of OHbG/QCMCS Hydrogels

The OHbG/QCMCS hydrogels were fabricated as previously described [82]. Briefly, QCMCS was dissolved in distilled water (DW) at a concentration of 3% (*w*/*v*). Lyophilized OHbG was also dissolved in DW at concentrations ranging from 3% to 11% (*w*/*v*). The QCMCS and OHbG solutions were mixed at a 2:1 volume ratio at room temperature. The gelation time needs 5 s after vortexing the QCMCS and OHbG solution for mixing. The resulting hydrogels were labeled OHbG/QCMCS 3, OHbG/QCMCS 5, OHbG/QCMCS 7, OHbG/QCMCS 9, and OHbG/QCMCS 11, corresponding to the OHbG concentrations.

### 4.6. Characterization

#### 4.6.1. Nuclear Magnetic Resonance Spectroscopy (NMR)

OHbG and QCMCS were prepared for measurement by dissolving the samples in D_2_O with a concentration of 10 mg/mL at 25 °C. The ^1^H NMR spectra were measured in 600 MHz using a Bruker AvanceIII-600 spectrometer (Bruker, Karlsruhe, German).

#### 4.6.2. Fourier Transform Infrared (FTIR) Spectroscopy

The FTIR spectra of OHbG/QCMCS hydrogels were measured by a FTIR spectrometer (TENSOR27, Bruker, Germany) after lyophilizing. The wavenumber range of about 4000–650 cm^−1^ was covered, with a resolution of 2 cm^−1^.

#### 4.6.3. Differential Scanning Calorimetry (DSC)

DSC was examined using Discovery DSC 2500 (TA Instruments, New Castle, DE, USA). A total of 10 mg of the sample was scanned at a rate of 10 °C/min under the nitrogen gas from 30 to 200 °C. The measurement was conducted under a nitrogen atmosphere.

### 4.7. Rheological Measurement

The rheological properties were evaluated using a Rheometer DHR2 (TA, New Castle, USA). The angular frequency sweep test was analyzed for measuring the storage modulus (G′) and loss modulus (G′′) of the hydrogel. Measurements were performed at a strain of 0.5% with a frequency starting at 0.1 rad/s and continuing up to 500 rad/s. The strain amplitude sweep tests with measurement storage modulus (G′) and loss modulus (G′′) of the hydrogel were conducted at 1.0 Hz, with a strain starting at 0.1% up to 1000%. The strain amplitude sweep tests for self-healing properties were measured at 1.0 Hz for repeated strains of 0.5% and 500% at a 100 s interval. All experiments were performed under 25 °C using 20 mm parallel plates.

### 4.8. Compressive Experiments

Compressive tests were performed using an Instron E3000LT (Instron Inc., Buckinghamshire, UK) at a speed of 10 mm/min on spherical hydrogels measuring 10 mm in height × 15 mm in diameter. All experiments were repeated three times.

### 4.9. Swelling Ratio Measurement

The swelling pattern of OHbG/QCMCS hydrogels was monitored at 37 °C with the following method. An equal amount of lyophilized OHbG/QCMCS hydrogel was kept in 40 mL of phosphate-buffered saline (PBS) with a pH 7.4 value to reach equilibrium. The swollen hydrogels were then weighed at preset time points. The swelling ratio was calculated by the following equation:

$$Swelling\;ratio\left(\%\right)=\frac{W_{t}-W_{0}}{W_{0}}\times 100$$

*W_t_* is the mass of the swollen hydrogel at a set time point, and *W*_0_ is the initial mass of the lyophilized hydrogel. All experiments were repeated three times.

### 4.10. Degradation Profiles

Each wet OHbG/QCMCS hydrogel was kept in 40 mL of PBS buffer (pH 2.0) with shaking at 100 rpm. The remaining weight of the hydrogels was measured at a predetermined time interval. The degradation rate was computed using the following equation:

$$Remaining\;weight\left(\%\right)=\frac{W_{t}}{W_{0}}\times 100$$

*W_t_* is the mass of the hydrogel measured at a predetermined time point, and *W*_0_ is the initial mass of the hydrogel before immersing in an acidic solution. All experiments were repeated three times.

### 4.11. Morphology Analysis

The morphology of the OHbG/QCMCS hydrogels was analyzed using FE-SEM (JSM-7800F Prime, JEOL Ltd., Akishima, Japan). Samples of OHbG/QCMCS 3, 5, 7, 9, and 11 were rapidly frozen at −71°C and then lyophilized. The dried samples were mounted onto double-sided carbon tape and coated with a thin gold layer under vacuum conditions to enhance electrical conductivity. Images were captured at 100× magnification using an excitation voltage of 5 kV.

### 4.12. Drug Release Study

The release behavior of 5-fluorouracil (5-FU) encapsulated in OHbG/QCMCS hydrogels was studied using two different pH conditions. Briefly, 1 mg/mL of 5-FU was dissolved in OHbG/QCMCS solution. Then, the 5-FU encapsulated hydrogels were incubated in 30 mL of acidic and physiological PBS buffer condition (pH 2.0 and pH 7.4) at 37 °C and shook at a 100 rpm. The measurements were conducted in pH 7.4 PBS buffer for 6 h, followed by a change to pH 2.0 PBS buffer to continue the experiment monitoring the pH-responsive release pattern. At predetermined time intervals, 500 μL of buffer containing the released drug was sampled, and an equal amount of buffer was replenished to maintain the total volume of the solution. Temperature and pH conditions were maintained during the releasing test. The concentration of the drug was calculated using the following equation:

$$The\;cumulative\;amount\;of\;5-FU=C_{n}V+\sum_{i=1}^{i=n-1}C_{i}V_{i}$$

*V* represents the total volume of the solution, *V_i_* is the volume of the sample, and *C_n_* and *C_i_* are the concentrations of the drug in the PBS testing solution and extraction sample. All experiments were repeated three times.

### 4.13. Antioxidant Efficiency

The antioxidant ability of OHbG/QCMCS hydrogels was evaluated by a DPPH radical scavenging assay [83]. A total of 50 mg of hydrogels was placed in 5 mL of 0.1 mM DPPH solution (EtOH:DW, 1:1) and incubated at 30 °C for 30 min in the dark. Thereafter, the absorbance at 517 nm of the DPPH solution was recorded using a SpectraMax Microplate Reader (Molecular Devices, Sunnyvale, CA, USA). The DPPH radical scavenging activity was quantified using the following equation:

$$DPPH\;radical\;scavenging\;activity\left(\%\right)=\frac{A_{0}-A_{s}}{A_{0}}\times 100\;\%$$

*A*_0_ is the absorbance of the DPPH in EtOH:DW 1:1 solution, and *A_s_* is the absorbance of the activated solution with the hydrogel. All experiments were repeated three times.

### 4.14. Antibacterial Activity Test

The antibacterial activity of the OHbG/QCMCS hydrogels was evaluated against *Escherichia coli* (*E. coli*) and *Staphylococcus aureus* (*S. aureus*), following a previously described method with slightly modifications [84]. Briefly, *E. coli* and *S. aureus* strains were cultured 18 h in Luria–Bertani broth (LB broth). Equal amounts of the hydrogel sample were co-incubated with 50 μL of bacterial suspension with a concentration of 10^7^ CFU/mL for 4 h. The control group was treated with the normal bacterial solution without adding any sample. After 4 h, 200 μL of the culture medium was added, respectively, to elute bacteria that had adhered to the surface of the hydrogel. Subsequently, 50 μL of the diluted bacterial suspension was spread on an agar plate. After being incubated at 37 °C for 24 h. The colonies formed on the agar plates were observed. All experiments were repeated three times.

### 4.15. In Vitro Cytotoxicity Test

Human embryonic kidney 293 (HEK-293) cells (Bank of Korea cell line, Seoul, Korea) were used to determine the in vitro cytotoxicity of OHbG/QCMCS hydrogels with the WST-8 assay [85]. Briefly, HEK-293 cells were cultured in Dulbecco’s minimum essential medium (DMEM, WELGENE, Seoul, South Korea), supplemented with 10% fetal bovine serum and 1% penicillin and streptomycin. A total of 5 mg of hydrogel sample was added to a 96-well culture plate (Costar, Cambridge, MA, USA) and incubated at 37 °C with 5% CO_2_. DMEM medium without treatment was used as a negative control, and DMEM medium containing 10% dimethyl sulfoxide (DMSO) was used as a positive control. After 48 and 72 h, 100 μL of the WST-8 assay reagent (QuantiMax, BIOMAX, Seoul, Republic of Korea) was added to each 96-well. Afterwards, cell viability was investigated by detecting the absorbance at 450 nm using a SpectraMax Microplate Reader (Molecular Devices, Sunnyvale, CA, USA). The percentage of cell viability was calculated with the following equation:

$$Cell\;viability\left(\%\right)=\frac{Absorbance\;of\;cells\;with\;OHbG/QCMCS\;hydrogels}{Absorbance\;of\;negative\;control\;cells}\times 100$$

All experiments were repeated three times.

## Data Availability

The original contributions presented in this study are included in the article. Further inquiries can be directed to the corresponding authors.

## Supplementary Materials

The following supporting information can be downloaded at: https://www.mdpi.com/article/10.3390/gels11030169/s1, Figure S1: Images of OHbG/QCMCS hydrogel in drug releasing test. Table S1: OHbG/QCMCS samples initial weight for swelling test.
