# Supplementary material for: Self-Healing Hydrogels with Intrinsic Antioxidant and Antibacterial Properties Based on Oxidized Hydroxybutanoyl Glycan and Quaternized Carboxymethyl Chitosan for pH-Responsive Drug Delivery
Source: Gels. 2025 Feb 26;11(3):169. doi: 10.3390/gels11030169 (PMC11942413; doi:10.3390/gels11030169)
Supplement: Supplementary file 1 [file gels-11-00169-s001.zip › gels-3480538-supplementary.pdf]

Self-Healing Hydrogels with Intrinsic Antioxidant and Antibacterial Properties Based on Oxidized Hydroxybutanoyl Glycan and Quaternized Carboxymethyl Chitosan for pH-Responsive Drug Delivery

Jae-pil Jeong [1,†], Kyungho Kim [1,†], Eunkyung Oh [1], Sohyun Park [1] and Seunho Jung [1,2,*]

[1] Department of Bioscience and Biotechnology, Microbial Carbohydrate Resource Bank (MCRB),
Konkuk University, 120 Neungdong-ro, Gwangjin-gu, Seoul 05029, Republic of Korea; jjp0531@naver.com (J.-p.J.); rudgh971225@naver.com (K.K.); eunkyung_5@naver.com (E.O.) so63991@naver.com (S.P.)

[2] Department of System Biotechnology, Microbial Carbohydrate Resource Bank (MCRB), Konkuk University, 120 Neungdong-ro, Gwangjin-gu, Seoul 05029, Republic of Korea

* Correspondence: shjung@konkuk.ac.kr; Tel.: +82-1032-372-669

† These authors contributed equally to this work.

Table S1. OHbG/QCMCS samples initial weight for swelling test.

	OHbG/QCMCS 3	OHbG/QCMCS 5	OHbG/QCMCS 7	OHbG/QCMCS 9	OHbG/QCMCS 11
Weight (mg)	28.25±0.91	32.36±0.84	38.50±0.79	43.57±1.12	47.39 ± 1.36

Each samples measured triplicated.

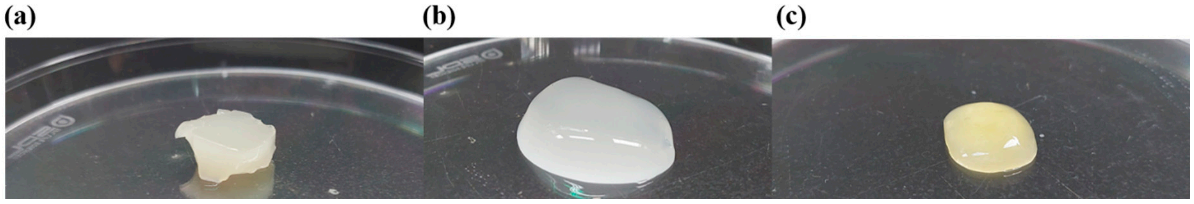

Figure S1. Images of OHbG/QCMCS hydrogel in drug releasing test. (a) Before releasing test. (b) After releasing at pH 7.4. (c) After releasing at pH 2.0
